# Therapeutic Fasting in Reducing Chemotherapy Side Effects in Cancer Patients: A Systematic Review and Meta-Analysis

**DOI:** 10.3390/nu15122666

**Published:** 2023-06-08

**Authors:** Yvelise Ferro, Samantha Maurotti, Maria Grazia Tarsitano, Oscar Lodari, Roberta Pujia, Elisa Mazza, Lidia Lascala, Raffaella Russo, Arturo Pujia, Tiziana Montalcini

**Affiliations:** 1 Department of Medical and Surgical Science, University Magna Grecia, 88100 Catanzaro, Italy; yferro@unicz.it (Y.F.); mariagrazia.tarsitano@unicz.it (M.G.T.); roberta.puj@gmail.com (R.P.); raffaella.russo.md@gmail.com (R.R.); pujia@unicz.it (A.P.); 2 Department of Clinical and Experimental Medicine, University Magna Grecia, 88100 Catanzaro, Italy; smaurotti@unicz.it (S.M.); oscar.lodari@outlook.it (O.L.); llascala1@gmail.com (L.L.); tmontalcini@unicz.it (T.M.); 3 Research Center for the Prevention and Treatment of Metabolic Diseases, University Magna Grecia, 88100 Catanzaro, Italy

**Keywords:** chemotherapy, chemotherapy toxicity, fasting, time-restricted eating, intermittent fasting

## Abstract

The aim of this study was to assess the available evidence regarding the effect of a variety of fasting-like regimens on preventing chemotherapy-related side effects. PubMed, Scopus and Embase were used to select the studies for this review, which concluded on 24 November 2022. All types of clinical trials and case series reporting chemotherapy toxicity associated with fasting regimens and any comparison were considered. A total of 283 records were identified, of which 274 were excluded, leaving only nine studies that met the inclusion criteria. Five of these trials were randomized. Overall, moderate to high-quality evidence showed that several fasting regimens did not provide benefits compared to a conventional diet or other comparators in reducing the risk of adverse events. The overall pooled estimate for a variety of fasting regime when compared to non-fasting, indicated no significant difference in the side effects (RR = 1.10; 95% CI: 0.77–1.59; *I*^2^ = 10%, *p* = 0.60), including neutropenia alone (RR = 1.33; 95% CI: 0.90–1.97; *I*^2^ = 0%, *p* = 0.15). A sensitivity analysis confirmed these results. Based on our systematic review and meta-analysis, there is currently no evidence supporting the superiority of therapeutic fasting over non-fasting in preventing chemotherapy toxicity. The development of cancer treatment that do not entail toxicities remains imperative.

## 1. Introduction

Chemotherapy is the primary treatment for most cancers. However, a large number of cancer patients who undergo chemotherapy experience mild to severe side effects [1]. The major chemotherapy-related side effects reported by various studies include nausea, vomiting, weakness, fatigue, loss of appetite, gastrointestinal symptoms, pain, and hematologic toxicity, which severely impact patients’ quality of life [2,3,4,5]. Despite advancements [6,7], inadequately controlled vomiting and nausea remain major problems in the several patients who delay or discontinue oncological treatments and consequently increase their mortality risk [8]. Therefore, in addition to improving survival rates, the development of non-toxic therapies in the cancer care setting is imperative. The conflicting evidence on the benefits of calorie restriction in the prevention of cancer cell malignancy and its detrimental effects has recently attracted great attention [9]. A variety of fasting regimens exist, with several preclinical studies suggesting that fasting, periodic fasting, and reducing calorie intake can prevent tumorigenesis, increase the efficacy of various chemotherapeutic agents, and prevent chemotherapy-related side effects [10,11,12,13,14]. However, contrary to these findings, other preclinical [14,15] and clinical studies [16,17,18,19,20] have found no evidence of therapeutic fasting protecting against tumors or improving chemotherapy efficacy. Based on all this evidence, fasting, although tumor-suppressive in healthy cells, may not exert beneficial effects in cancer cells [21,22,23,24,25]. However, a variety of fasting regimens seem to be capable of preventing the undesirable effects of chemotherapy [16,17,18]. It is well known that certain chemotherapeutic agents lead to anemia, neutropenia, and/or thrombocytopenia. As a result, treatment doses are lowered to reduce myelosuppression, infection risk, and febrile neutropenia [26]. Reduced mortality following an infection has been demonstrated in previously fasted mice, while force-feeding infected mice dramatically increased their mortality rates [27]. Several fasting regimes can reverse chemotherapy-induced immunosuppression through hematopoietic stem cell-based regeneration [28]. It has been reported that fasting decreases IGF-1 levels to direct energy expenditure from growth to survival, promoting apoptosis in damaged cells and contributing to an extended life span [29]. Furthermore, chemotherapy-induced gastrointestinal toxicity likely occurs when the gastrointestinal mucosal barrier is disrupted, leading to the release of pro-inflammatory cytokine and mucositis [30]. In animal models, cycles of fasting and re-feeding have been shown to reduce lymphocyte numbers, intestinal infiltration, increase intestinal regeneration, and lower multiple markers of systemic inflammation, partially through modulation of the gut microbiome [31]. However, only a few small clinical studies have evaluated the potential of these dietary regimens as adjuncts to chemotherapy in reducing its toxicity. Furthermore, it remains unclear whether fasting regimens in cancer patients predispose to cachexia or worsen malnutrition.

The objectives of this systematic review and meta-analysis are to determine the strength of the evidence regarding the effect of various fasting-like regimens in cancer patients undergoing chemotherapy treatments, specifically in preventing chemotherapy-related side effects, and to evaluate their safety and tolerability.

Unfortunately, the application of fasting and fasting-mimicking diets (FMDs) in cancer patients has been prematurely reported by the media and promoted at scientific meetings as a potential therapeutic option in medical oncology. If the available evidence supports the utility of FMD regimens, this work may contribute to a better understanding and a clear step towards implementing fasting regimens during chemotherapy for patients with cancer, either as a safe and effective alternative or as an adjunct to current standard treatments. Alternatively, this work will emphasize the need for extreme caution when considering the clinical applications of fasting. It has the potential to have far-reaching consequences for cancer treatment in general.

## 2. Materials and Methods

A systematic approach for searching and reviewing literature was used in accordance with the Preferred Reporting Items for Systematic Reviews and Meta-Analyses (PRISMA) guidelines [32]. The electronic databases PubMed (MEDLINE), Scopus, and Embase were used to select the studies included in this systematic review and meta-analysis. The search for articles concluded on the 24 November 2022. A combination of the following keywords was used to search for relevant literature: (‘chemotherapy’, ‘chemotherapy toxicity’, ‘chemotherapy-related side effects’) for the population; (‘fasting’, ‘fasting-mimicking diet’, ‘short-fasting’ ‘time-restricted eating’, ‘intermittent fasting’) for the intervention.

Intermittent fasting (IF) refers to episodic periods of little or no calorie intake. It encompasses a variety of programs that manipulate the timing of eating occasions by incorporating short-term fasting (STF), which involves food intake within a short period. Different patterns include fasting every other day, complete 24-h fasting, or fasting on one or two nonconsecutive days per week. Many fasting programs recommend no or limited caloric intake (≤500 kcal/day) during the fasting period, combined with an unlimited number of calorie-free beverages (i.e., water or coffee). Fasting-mimicking diets (FMDs) are specific meal plans formulated to simulate the fasting state. Specific diets include a variety of plant-based foods. Unlike fasting, FMDs provide vitamins and minerals.

The protocol was prospectively registered at the International Prospective Register of Systematic Reviews (PROSPERO) registry (CRD42022378131); https://www.crd.york.ac.uk/prospero/display_record.php?ID=CRD42022378131 (accessed on 3 December 2022).

### 2.1. Study Selection Process

The inclusion criteria were as follows: (1) studies reporting chemotherapy-related toxicity in cancer patients undergoing therapeutic fasting or fasting-like regimens; (2) all types of clinical trials and case series, irrespective of participants’ age and gender; (3) a minimum sample size of *n* = 10 (participants). Although the language was restricted to English, no cultural restrictions were placed on eligible studies during the search. The exclusion criteria included pre-clinical studies, epidemiological studies, and incidence and prevalence studies. Human studies involving patients who were not receiving standard care for their malignancies were also excluded.

### 2.2. Data Extraction and Quality Assessment

The search results were imported into the reference management software RefWorks (ProQuest) to store, organize, and avoid duplicates.

The abstracts found to be relevant to the topic of interest were shortlisted by two researchers (OL and YF). Full-length papers of the selected articles were assessed against the eligibility criteria. The articles that fulfilled the inclusion criteria were included in the final systematic review. Any disagreements between the authors were resolved through discussion until a consensus was reached; otherwise, a senior author (TM) arbitrated. To minimize bias and random error, two researchers rigorously evaluated the allocation, randomization, blinding, comparability of the treatment and control groups, appropriateness, the quality of the analysis performed, and the protocol registration and pre-specification of inclusion criteria.

An electronic data sheet was developed and used for data extraction. We extracted details on the population characteristics (e.g., cancer patients, type of cancer, sample size), the intervention and comparator (e.g., type of intervention such as FMD, STF, IF, and any comparison), and the outcomes. The data were summarized by all reviewers in a graphical summary. The research questions were drawn using the PICO framework, which specifies the five key components of a well-defined therapeutic question: population, intervention, comparison, outcomes, and study design (Table S3). We provided a narrative description of the PICOs stratified into three categories: the “beneficial” category was assigned if there was evidence of a positive effect, the “harmful” category was used when the conclusions indicated a harmful effect, and the “no differential effect” category was applied when the conclusions indicated no difference between the intervention and the comparator.

CASP (Critical Appraisal Skills Programme) checklists were used as a tool for quality appraisal in health-related qualitative evidence syntheses [33]. The studies did not undergo critical appraisal because the inclusion criteria required a specific study design; thus, methodological differences were not present. If the data from the same sample were presented in separate studies, the study with the largest sample size or the study examining more than one side effect was selected. If published data were insufficient to determine an effect size, the study was excluded.

Bias due to missing results was avoided by consulting multiple bibliographic databases, trials registers, study authors, or sponsors. The funnel plot was used as graphical device to assess study precision and systematic heterogeneity (Figure S1).

### 2.3. Definition of Outcomes

The primary outcomes were Adverse Events (AEs) higher than grade II (considering AEs all together) and Neutropenia, according to the Common Terminology Criteria for Adverse Events (CTCAE) as reported in each study. We used a common summary outcome measure.

### 2.4. Data Synthesis and Analysis

Dichotomous data were analyzed by calculating the risk ratio (RR) for each trial with 95% confidence intervals (CIs). We assessed the heterogeneity of trial results using the Chi-square test of heterogeneity and the *I*^2^ measure of inconsistency. The main analysis was conducted using random-effects models. The heterogeneity was measured using the *I*^2^ statistic, with values greater than 50% indicating substantial heterogeneity [34]. We performed a meta-analysis using Cochrane’s Review Manager (RevMan) Software version 5.4. The pooled effect estimate from the meta-analysis was used to assess the certainty in evidence. The GRADE (Grading of Recommendation, Assessment, Development and Evaluation) approach was used to assess the outcome-specific certainty of the evidence in systematic reviews [35]. The GRADE approach classifies bodies of evidence as high, moderate, low or very low. Statistical significance was set at *p* < 0.05.

## 3. Results

### 3.1. Search Results

**Figure 1:**
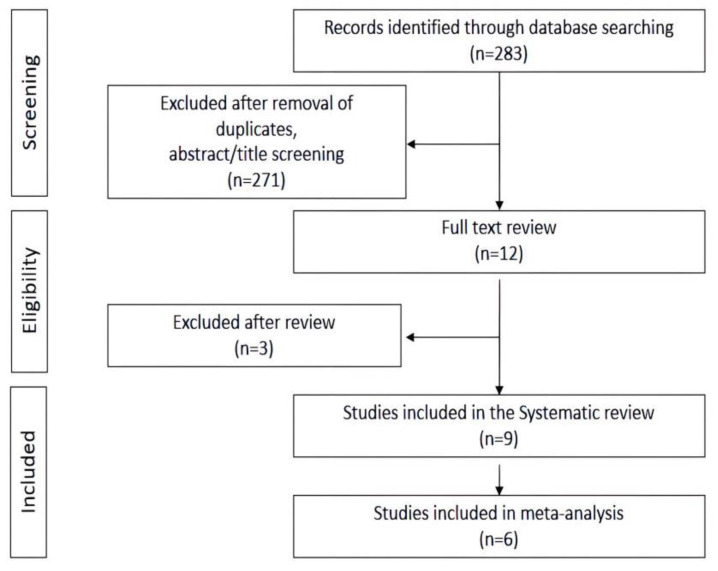
PRISMA flowchart for the selection of studies.

A total of 283 unique records were identified through the database and bibliographic searches. Of these, 274 were excluded based on the title, abstract content, and full texts; thus, only nine records met inclusion criteria (a flow diagram of the review process is shown in Figure 1).

### 3.2. Risk of Bias

The methodological quality of the studies is shown in Table S1. A total of four studies were rated as having a low risk of bias [16,18,36,37,38]. The allocation concealment and selective reporting of these studies were appropriate. All studies had no risk of bias in terms of incomplete reporting. The overall quality of the studies was assessed, and three were rated as having a high risk of bias [16,17,18,36,37,38,39]. There were two separate studies that presented data from the same sample [37,40]; therefore, only the study that examined more side effects were included in the analysis [37].

### 3.3. Study Characteristics

In six single-center trials [16,17,18,36,37,38,39], FMD, STF, and IF (consisting only of clear liquids such as water, broth, tea, and coffee) were tested for a minimum of two cycles of chemotherapy. In six trials [16,17,18,36,38,39] these dietary regimens were tested before and after chemotherapy. Only five of these trials were randomized [16,18,36,37,38], and overall, a trend in reduction of AEs such as headache, nausea, vomiting, diarrhea, and hematologic toxicity (neutropenia) was reported with fasting-like regimens compared to controls. In all these trials, malnourished patients were excluded (based on low body mass index-BMI), and standard antiemetics were administered. Although standard chemotherapy protocols were adopted, none of these RCTs reported whether a granulocyte colony-stimulating factor (G-CSF) prescription was allowed to reduce neutropenic complications. Double blinding was not feasible in these types of RCT. Therapeutic fasting (in the form of IF, STF, and FMD) for a period of 24–72 h before chemotherapy and/or 24 h following chemotherapy in patients with cancer was well tolerated without any serious side effects. No patients experienced significant weight loss at the end of the studies. One study reported that IF throughout the day for three consecutive days with a restriction of sugar and fat was safe and feasible, and a greater reduction of diarrhea, vomiting, and nausea was observed compared to the non-fasting regimen [38]. However, a higher incidence of constipation was reported in IF patients compared to non-fasting patients [38].

In four studies [16,18,36,37], the energy intake from the diet was less than 350 kcal/day, and in the study by Omar [38], it was up to 750 kcal/day. Four studies [16,18,36,37,38,39] reported that less than 60.0% of the patients on fasting-like regimens complied during all cycles of chemotherapy. STF protected against a chemotherapy-dependent reduction in erythrocyte counts and prevented DNA damage in healthy cells compared to the non-fasted group [16].

Only the DIRECT study [37] an open label, randomized study designed to evaluate the impact of an FMD on toxicity, as well as on the radiological and pathological response to chemotherapy for breast cancer, was a multicenter RCT. In this study, 65 participants received FMD as an adjunct to chemotherapy, while 64 participants used their regular diet. The participants were randomized in a 1:1 ratio to receive either the FMD or regular diet for three days prior to and on the day of each cycle of chemotherapy. The FMD consisted of a four-day plant-based liquid diet with low amino-acid substitution (including soups, broths, tea, and other liquids). The calorie intake was ~1200 kcal per day for the first day, followed by ~200 kcal for the next three days (with complex carbohydrates providing >80% of the energy). Similar to other RTCs, the primary endpoint of the DIRECT study was grade III/IV toxicity. The authors found that toxicity, assessed throughout all cycles of chemotherapy, was not significantly different between the FMD patients and the regular diet group (75.4% vs. 65.6%, respectively), despite the FMD patients not receiving dexamethasone treatment.

In the per-protocol analysis, chemotherapy-related toxicity did not differ between FMD-compliant patients and the control group. As expected, urine ketone bodies were higher in the FMD group compared to the control group. Quality of life (assessed by QoL) was a secondary outcome in the DIRECT trial. The QoL was not significantly different between the groups. The DNA damage after chemotherapy was significantly lower in CD45+ CD3+ T-lymphocytes from participants on the FMD compared to those on a regular diet (*p* = 0.045). Only 20.0% of the patients on the FMD complied with the diet throughout all cycles of chemotherapy, and 85% of FMD patients reported unpleasant taste as the reason for discontinuing the treatment.

None of the interventions tested resulted in beneficial effects (according to the main outcomes), and all were classified as having “no differential effect” (Table 1).

Overall, moderate/high-quality evidence indicates that several fasting regimens did not demonstrate any benefits compared to a conventional diet or other comparators in reducing the risk of AEs (Table S1).

In a case-series study [19] involving a heterogeneous group of patients with cancer, including those with breast, prostate, ovarian, uterus, lung, and esophageal cancer, who voluntarily fasted, STF was found to be safe and well-tolerated during six cycles of chemotherapy. It also reduced some side-effects such as GI issues, fatigue, and weakness.

### 3.4. Fasting Regimes and the Risk of Chemotherapy-Side Effects

**Figure 2:**
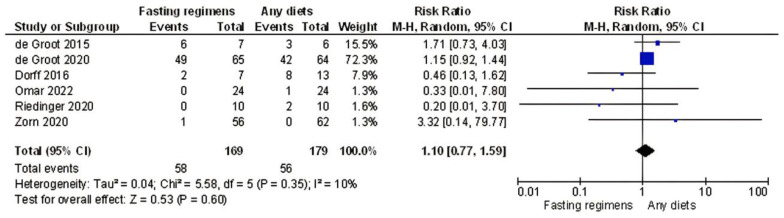
Forest plot for total chemotherapy-related toxicity (Grade > 2). de Groot et al. [16], de Groot et al. [37], Dorff et al. [17], Omar et al. [38], Riedinger et al. [36], Zorn et al. [39]; CI, confidence interval. *I*^2^ measure of heterogeneity.

Since there were no grade III/IV toxicity events documented in the study of Bauersfeld et al. [18], and in two studies [16,36,37,38], none of the participants developed neutropenia, we excluded these works from the estimation of the risk of chemotherapy side effects in the overall pooled estimate and the neutropenia pooled estimate, respectively (Figure 2 and Figure S2).

The overall pooled estimate for a variety of fasting regimes relative to a non-fasting regime suggested no significant difference in the risk of chemotherapy side effects from six single-center trials (RR = 1.10; 95% confidence interval [CI]: 0.77–1.59; *I*^2^ = 10%, *p* = 0.60; Figure 2). These results were consistent even when considering only neutropenia (four trials, RR = 1.3395% confidence interval [CI]: 0.90–1.97; *I*^2^ = 0%, *p* = 0.15, Figure S2) [16,30,31,32].

### 3.5. Sensitivity Analysis

**Figure 3:**
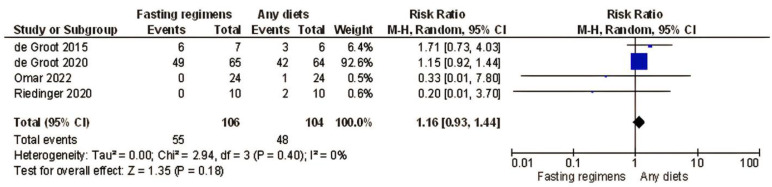
Forest plot for total chemotherapy-related toxicity (Grade > 2)—Sensitivity analysis. de Groot et al. [16], de Groot et al. [37], Omar et al. [38], Riedinger et al. [36]; CI, confidence interval. *I*^2^ measure of heterogeneity.

**Figure 4:**
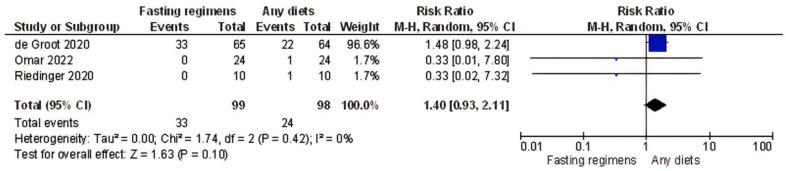
Forest plot for chemotherapy-related toxicity: neutropenia (Grade > 2) sensitivity analysis. Groot et al. [37], Omar et al. [38], Riedinger et al. [36]; CI, confidence interval. *I*^2^ measure of heterogeneity.

The sensitivity analysis, excluding the non-randomized trials [17,39] resulted in no differences in the odds ratios and significance of results with regard to the pre-specified endpoint (in four RCT, total toxicity: RR = 1.16; 95% confidence interval [CI]: 0.93–1.44; *I*^2^ = 0%, *p* = 0.18, Figure 3; in three RCT, neutropenia RR = 1.40, 95% confidence interval [CI]: 0.93–2.11; *I*^2^ = 0%, *p* = 0.10, Figure 4).

## 4. Discussion

Fasting for disease prevention, particularly in the context of oncology, has recently become a popular topic [9]. Due to the potential risks associated with this approach, it is important to evaluate whether the hypothesized benefits of a variety of fasting regimens can truly affect clinical oncologic endpoints. This review compiled data from 348 patients with cancer across nine studies on chemotherapy-related toxicity. Our systematic review did not show any clear effect of several fasting-like regimens in reducing chemotherapy-related side effects such as nausea, vomiting, weakness, and hematologic toxicity (analyzed collectively, see Table 1), regardless of the cancer type, duration, and type of intervention, which severely impact the quality of life of cancer patients undergoing chemotherapy.

The current meta-analysis, to the best of our knowledge, is the first to demonstrate that therapeutic fasting (STF/FMD/IF; patients *n* = 210) is not superior to non-fasting in preventing chemotherapy-related side effects when evaluating both randomized and non-randomized studies together (Figure 3 and Figure 4). After a quality appraisal, we included only four RCTs in the sensitivity analysis. We did not find a significant difference in the rate of AEs (grade III/IV) and neutropenia (only three RCT) between participants on fasting-like regimens and non-fasting individuals (*p* = 0.18 and *p* = 0.10, respectively, Figure 3 and Figure 4), although individual studies reported lower rates of AEs for those on fasting-like regimens [16,18,36,37,38]. The reason for planning a pooled analysis of AEs was to provide a more statistically robust estimate of the effect of a fasting-like diet on outcomes that the individual AEs had limited power to demonstrate. In all these trials, standard antiemetics were allowed for all patients, ensuring that the control group matched the treatment group.

The results from this review are comparable to work by Caccialanza et al. [9], which synthesized most of the available studies regarding fasting or calorie restriction in cancer patients. Fasting or calorie restriction did not consistantly show anticancer effects in preclinical studies [10,11,12,13,14,15]. Furthermore, the evidence provided by preclinical and human studies on the beneficial effect of a variety of fasting regimens on chemotherapy-related side effects is still very limited.

Despite differences in term of calories intake, STF, or periodic fasting in general, FMD and IF achieve their beneficial effects through mechanisms that are independent of reduced calorie intake [41]. For these reasons, and also due to the fact that only a few clinical studies have evaluated the effect of these dietary regimens on cancer patients, we analyzed the overall findings of the studies.

Despite their high quality, only a few clinical studies have evaluated the potential of fasting, STF, FMD, and IF to improve the management of chemotherapy-related side effects, which leads to the false impressions about the magnitude and existence of an effect [42]. These dietary regimens seemed to be potentially effective during chemotherapy treatment in reducing symptoms of nausea, vomiting, fatigue, and hematologic toxicity [16,17,18,36,37,38,39,40]. However, the majority of studies were single-institutional trials [16,17,18,36,38,39], and only four RTC were carried out [16,36,37,38]. Given the scarcity of literature exploring this theme and the negative (but not definitive) findings by the current meta-analysis, it is not possible to draw a conclusion on the effectiveness of fasting during chemotherapy. All these clinical studies showed only that fasting as an adjunct to chemotherapy is safe and may be well tolerated, but not in all patients. These studies remain the core of the limited evidence on this topic to date.

The DIRECT study [37], the only multicenter RCT reported in the literature, primarily suggests that with an FMD regimen, there is no need for dexamethasone in the prevention of chemotherapy side effects. Notably, in the study, a, FMD kit, which is now commercialized on the web, was used. It is possible that 24–72 h of fasting before chemotherapy and 24 h following chemotherapy may be not sufficient to reduce the side effects that occur during chemotherapy treatment. However, it has been reported that less than 40.0% of patients on a fasting regimen complied during all cycles of chemotherapy [16,18,36,39]. Due to the fact that it would be extremely difficult to restrict patients in terms of calorie intake for many days, fasting (or other fasting regimes) does not seem to be a promising strategy to increase the tolerability of chemotherapy in cancer patients. In the DIRECT trial [37], chemotherapy-induced DNA damage in T lymphocytes was significantly reduced in patients on the FMD regimen. Furthermore, patients in the FMD cohort were more likely to have a high rate (90% to 100%) of tumor cell loss after neoadjuvant chemotherapy. This is the only study on fasting that considered a pathologic outcome with prognostic information. Such pathologic endpoints provide early indicators of therapy response and represent an efficient clinical trial design for evaluating the efficacy of novel neoadjuvant therapies. Nevertheless, questions have been raised over the appropriateness of these pathologic endpoints as surrogates for long-term clinical outcomes. More well-designed RCTs are needed to assess the effects of FMD on recurrence risk and survival outcomes before recommending the practice of fasting or FMD in patients with cancer [43].

Furthermore, not all cancer patients can undergo fasting. Several conditions, such as a very low body mass index and malnutrition, are incompatible with therapeutic fasting. People with cancer are among the most malnourished of all patient groups [44,45]. Daily energy intake during hospitalization is an independent predictor of all-cause mortality after discharge in patients at risk of malnutrition [46,47]. It is well accepted that a low energy intake for a brief period (such as during hospitalization) and at the time of discharge can worse cachexia-induced systemic inflammation [47], which is associated with anorexia, appetite loss, nausea, and taste disorders [48]. Patients with cachexia experience severe weakness and fatigue with increased chemotherapy toxicity [49] and decreased efficacy [50]. All of these concepts contradict the hypothesis that fasting improves clinical outcomes in cancer patients. None of the patients experienced significant weight loss at the end of the studies. However, malnourished patients were excluded from RCTs. Tus, FMD regimens are safe only in patients with a normal BMI.

Moreover, during calorie restriction, the glucose concentration declines, and the stored glycogen becomes the main energy source. Once the glycogen is depleted, the organism utilizes glycerol and fatty acids mobilized from the adipose tissue [51]. Ketone bodies thus increase [37] and may become the main fuel. High blood levels of ketones may cause loss of appetite, nausea, and vomiting [52]. These mechanisms may worsen the clinical picture of chemotherapy adverse events rather than improve it.

### 4.1. Limitations of the Study

The results of this meta-analysis should be interpreted cautiously due to the variability in sample sizes among studies. Assessing the contribution of age, co-morbidities, and polypharmacy to the outcome remains difficult. Studies that were not published in English were excluded. Nutrition research conducted to examine the role of diet in cancer is particularly challenging, as it involves assessing and intervening in patients’ lifestyles, which are influenced by their cancer diagnosis. Another challenge lies in the collection of data (data are largely self-reported) and how the results are described. Thus, nutrition research results are often misinterpreted. Moreover, participants enrolled in feeding trials face a significant burden, and the risk for nonadherence is high. Improved treatment outcomes depend on adherence to the prescribed diet. Compliance with FMD regimes in combination with chemotherapy remains a major issue. In patients who fasted before and after chemotherapy, no major side effects caused by fasting itself, other than hunger and lightheadedness, were reported. In the DIRECT trial [37] less than 40.0% of the patients complied with a FMD regimen during all cycles of chemotherapy, and in the trial by Zorn et al. [39], only approximately half expressed a willingness to fast again during chemotherapy. Enhancing patient education, understanding, and engagement may play a key role in improving compliance with FMD regimes. Understanding the perceived facilitators or barriers to the adherence to FMD will likely result in increased compliance and improved clinical outcomes with FMDs [53]. However, the inclusion of qualitative literature enriches the data synthesis. We included RCTs that assessed short-term dietary exposure. Feeding trials are not suitable for answering questions about longer-term dietary exposures. Since the studies included in our work were sufficiently homogeneous in terms of subjects, interventions, and outcomes, we performed a meta-analysis to provide a meaningful summary. In a recent work by Drexler U. et al. [54], a meta-analysis was not conducted. We assessed the heterogeneity of the included studies using the I-squared statistic and found a low I-squared value (0%), indicating that a meta-analysis was an appropriate measure to express the summary findings. Unlike the review by Drexler U. et al. [54], we included only studies that compared pairs of interventions (FMD versus conventional diet). In the study by Lende, T. H. [55], the control group received a carbohydrate-based ONS nutrition instead of a standard/usual diet. Therefore, we excluded the work of Lende, which was included in the review by Drexler et al.

Taken together, these attributes primarily render the conclusions derived from our meta-analysis as hypothesis-generating rather than definitive and/or conclusive. It cannot be excluded that in the future, if larger studies are carried out, completely different results from those reported here may be obtained (see the list of ongoing trials about fasting regimens in oncological patients, Table S2).

Considering the ongoing challenges with poor compliance to fasting regimens, future well-designed clinical trials should be carried out to determine if periodic fasting may provide benefits only patients with cancers associated with weight gain (such as breast, ovarian, and prostate cancer) rather than cachexia.

### 4.2. Implications for Clinical Practice

One must be cautious in the application of the clinical trials testing the effects of fasting or other fasting regimes in preventing chemotherapy-related side effects in cancer patients. The careful analysis of these clinical trials did not show any clear association between fasting regimens and the reduction of common side effects such as nausea, vomiting, weakness, gastrointestinal issues, and hematologic toxicity. We would argue that the role of fasting as a useful mechanism in reducing chemotherapy side effects remains unresolved, and much of the current debate may be unnecessary [9]. In fact, there if insufficient scientific evidence to support the prescription of food restriction before and/or after chemotherapy by oncologists or nutritionists. This review opens up a discussion on an area that has received little attention in the literature thus far, and these findings may serve as potential themes for future research. Future studies examining the pathogenesis and effects of treatment are urgently needed.

Although there is limited evidence regarding the association between chemotherapy-related side effects and general dietary recommendations, it is likely that an individualized approach to counselling, taking into account patients’ preferences, symptoms, and medical status, is a key element in improving the side effects of chemotherapy [56,57].

## 5. Conclusions

This systematic review revealed a lack of an evidence-based approach despite the existence of a misconception that fasting-like diets can mitigate treatment side effects. Therefore, further investigated is warranted. Due to the scarcity of literature on this topic, it is not possible to confirm the effectiveness of therapeutic fasting during chemotherapy. This finding highlights the unmet needs of cancer patients undergoing chemotherapy, emphasizing the need for for continued research and the development of tailored dietary/nutrition programs to assist them in managing treatment-related side effects. It is hoped that further large, randomized studies will help clarify the potential benefits of therapeutic fasting in specific patient populations and provide guidance on its implementation.

## Figures and Tables

**Table 1:** Characteristics of studies included in the systematic review.

Source	Study Size	Study Design	Cancer Type	Intervention Treatment	Comparison	Outcomes	Intervention Duration	Results	Conclusions
Riedinger et al. [30] 2020	24	RCT	Ovarian Uterine Cervical	STF 48 h— only water for 24 h before and 24 h following each chemotherapy cycle.	No dietary modification (balanced, normo-caloric diet).	Chemotherapy-related side effects and QOL.	6 cycles	There was no significant difference in chemotherapy-related side effects or in mean QOL scores between the groups.	No differential effect
de Groot et al. [16] 2015	13	RCT	Breast	STF 48 h— only water, coffee, or tea without sugar for 24 h before and 24 h following each chemotherapy cycle.	Normal diet according to the guidelines for healthy nutrition, with a minimum of two pieces of fruit per day.	Feasibility and chemotherapy-related side effects.	6 cycles	STF during chemotherapy was tolerated and reduced hematological toxicity (erythrocyte and thrombocyte counts). Non-haematological toxicity did not differ between the groups.	No differential effect
de Groot et al. [37] 2020	131	RCT	Breast	FMD 96 h—plant-based substitution diet with low amino acids consisting of soups, broths, liquids, and tea for three days prior to and on the day of each chemotherapy cycle.	Normal diet.	Chemotherapy-related side effects, toxicity, Radiological, and pathological response to chemotherapy.	6–8 cycles	There was no significant difference in chemotherapy-related side effects between the groups. A radiologically complete/partial response and pathological response occured more often in patients using the FMD. FMD also significantly reduced chemotherapy-induced DNA damage in T lymphocytes.	No differential effect
Omar et al. [38] 2022	48	RCT	Breast	IF 72 h— fasting for 18 h, from 12 a.m. to 6 p.m., with eating allowed for 6 h, from 6 p.m. to 12 a.m. Water consumption is permitted during fasting Hours, and small quantities of vegetables, fruits, proteins, and carbohydrates, are allowed, with limitated sugar and fats on the day before, during, and after chemotherapy.	Normal diet.	Safety, practicality, chemotherapy-related side effects, and toxicity.	4 cycles	IF during chemotherapy was tolerated and reduced the toxicity of chemotherapy to the gastrointestinal tract (nausea, vomiting, diarrhea, Grade I/II) compared to a normal diet. There was no significant difference in hematological parameters between the two groups after cycle 4.	No differential effect
Bauersfeld et al. [18] 2018	50	RCT	Breast Ovarian	STF 60 h—only water, herbal tea, and small, standardized quantities of vegetable juice and light vegetable broth for 36 h before and 24 h after each chemotherapy cycle.	Normocaloric Mediterranean diet.	Safety and QOL	4–6 cycles	STF during chemotherapy was tolerated and appeared to improve QOL and fatigue during chemotherapy (only AEs Grade I/II).	No differential effect
Dorff et al. [17] 2016	20	non-RCT	Breast Ovarian Uterine Urothelial NSCLC	STF 72 h—only water, non-caloric beverages, and small quantities of juice or food (under 200 kcal/24 h) for 48 h before and 24 h after chemotherapy.	STF 24 h or 48 h—only water, non-caloric beverages, and small quantities of juice or food under 200 kcal/24 h for 24 h or 48 h before chemotherapy.	Safety, feasibility, and chemotherapy-related side effects toxicity.	2 cycles	Reduction of DNA damage in leukocytes from subjects who fasted for ≥48 h (*p* = 0.08). There was no significant difference in neutropenia (Grade III/IV) in the 48 and 72 h cohorts compared to 24 h cohort.	No differential effect
Zorn et al. [39] 2020	51	non-RCT	Breast Endometrial Ovarian Cervical	mSTF 96 h—Ketogenic diet that provided between 400 and 600 kcal/day for 72 h before and 24 h after chemotherapy	Normocaloric diet	Chemotherapy-related side effects, toxicity, QOL, fasting-related discomfort, compliance, nutritional status, and laboratory parameters.	4–6 cycles	Reduced chemotherapy-induced toxicities (stomatitis, headaches, weakness; Grade I/II) and total toxicity score in mSTF group. There were significantly fewer post-mSTF chemotherapy postponements.	No differential effect
Lugtenberg et al. [40] 2021	131	RCT	Breast	FMD 96 h—plant-based, low amino acid substitution diet consisting of soups, broths, liquids, and tea) for three days prior to and on the day of each chemotherapy cycle.	Normal diet.	QOL and illness perceptions.	6–8 cycles	Improved QOL and illness percepition.	No differential effect
Sadfie et al. [19] 2009	10	Case series	Breast Prostate Ovarian UterineNSCLC, Esophageal	Voluntarily fasted 48–140 h and/or following 5–56 h prior to chemotherapy	/	Chemotherapy-related side effects, toxicity	Average of 4 cycles	Six patients during chemotherapy with or without fasting reported a reduction in fatigue, weakness, and gastrointestinal side effects while fasting.	No differential effect

RCT, randomized control trial; STF, short-term fasting; mST, modified short-term fasting; FMD, fasting-mimicking diet; IF, intermittent fasting; QOL, quality of life; NSCLC, non-small-cell lung cancer; AEs, Adverse events.

## Data Availability

The data are available from the authors upon request.

## Supplementary Materials

The following supporting information can be downloaded at: https://www.mdpi.com/article/10.3390/nu15122666/s1, Figure S1: Funnel Plot; Figure S2: Forest plot for chemotherapy-related toxicity—Neutropenia (Grade > 2); Table S1: Risk of bias assessment according to Critical Appraisal Skills Programme (CASP) for assessing randomized controlled trials and GRADE approach for assessing the certainty in evidence. Table S2: Ongoing Clinical trials about Fasting regimens in cancer patients. Table S3: PRISMA Checklist.
