# Supplementary material for: Therapeutic Fasting in Reducing Chemotherapy Side Effects in Cancer Patients: A Systematic Review and Meta-Analysis
Source: Nutrients. 2023 Jun 8;15(12):2666. doi: 10.3390/nu15122666 (PMC10303481; doi:10.3390/nu15122666)
Supplement: Supplementary file 1 [file nutrients-15-02666-s001.zip › Figures S1 and S2.pdf]

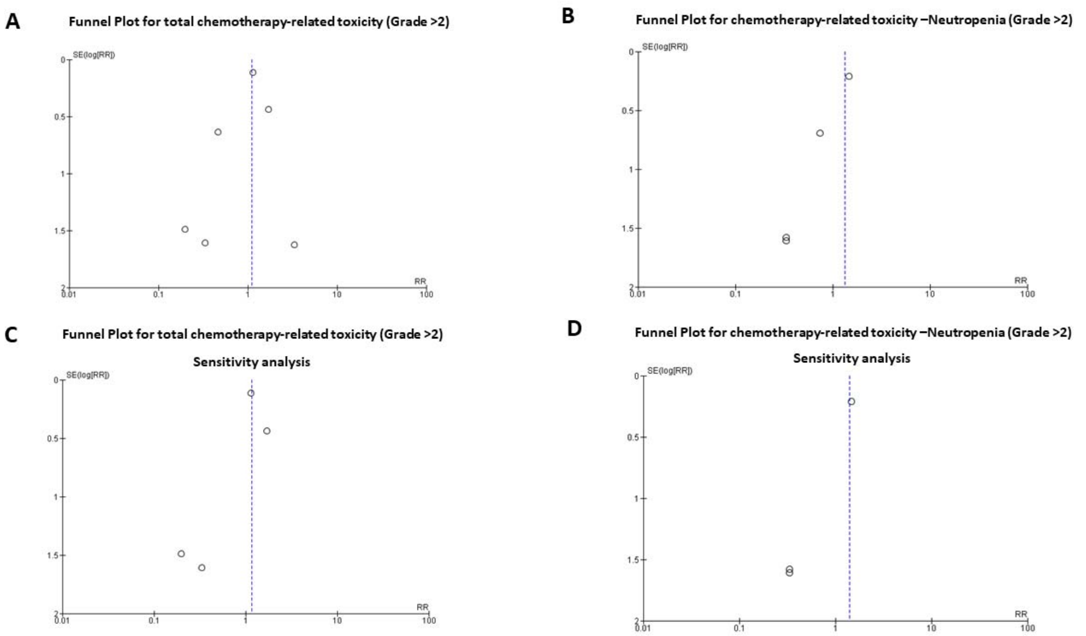

Figure S1. Funnel Plot.

Study or Subgroup	Fasting regimens Events	Fasting regimens Total	Any diets Events	Any diets Total	Weight	Risk Ratio M-H, Random, 95% CI
de Groot 2020	33	65	22	64	88.6%	1.48 [0.98, 2.24]
Dorff 2016	2	7	5	13	8.3%	0.74 [0.19, 2.89]
Omar 2022	0	24	1	24	1.5%	0.33 [0.01, 7.80]
Riedinger 2020	0	10	1	10	1.6%	0.33 [0.02, 7.32]
**Total (95% CI)**		**106**		**111**	**100.0%**	**1.33 [0.90, 1.97]**
Total events	35		29			

Heterogeneity: Tau² = 0.00; Chi² = 2.52, df = 3 (P = 0.47); I² = 0%
Test for overall effect: Z = 1.44 (P = 0.15)

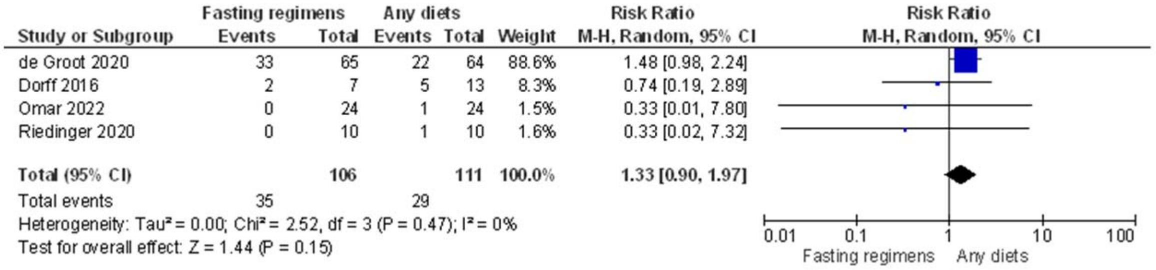

Figure S2. Forest plot for chemotherapy-related toxicity –Neutropenia (Grade >2)
