# Supplementary material for: Therapeutic Fasting in Reducing Chemotherapy Side Effects in Cancer Patients: A Systematic Review and Meta-Analysis
Source: Nutrients. 2023 Jun 8;15(12):2666. doi: 10.3390/nu15122666 (PMC10303481; doi:10.3390/nu15122666)
Supplement: Supplementary file 1 [file nutrients-15-02666-s001.zip › Table S1.pdf]

Table S1. Risk of bias assessment according to Critical Appraisal Skills Programme (CASP) for assessing randomized controlled trials and GRADE approach for assessing the certainty in evidence

Criteria	Riedinger et al, 2020	de Groot et al, 2015	de Groot et al, 2020	Omar et al, 2022	Bauersfeld et al, 2018	Dorff et al, 2016	Zorn et al, 2020
Did the study address a clearly focused research question?	Yes	Yes	Yes	Yes	Yes	Yes	Yes
Was the assignment of participants to interventions randomised?	Yes	Yes	Yes	Yes	Yes	No	No
Were all participants who entered the study accounted for at its conclusion?	Yes	Yes	Yes	Yes	Yes	Yes	Yes
Were the participants 'blind' to intervention they were given?	No	No	No	No	No	No	No
Were the investigators 'blind' to the intervention they were giving to participants?	Can't tell[a]	Can't tell[a]	Yes	Can't tell[a]	Can't tell[a]	No	No
Were the people assessing/analysing outcome/s 'blinded'?	Can't tell[a]	Can't tell[a]	Yes	Can't tell[a]	Can't tell[a]	No	No
Were the study groups similar at the start of the randomised controlled trial?	Yes	Yes	Yes	Yes	No	No	No
Apart from the experimental intervention, did each study group receive the same level of care (that is, were they treated equally)?	Yes	Yes	Acceptable	Yes	Yes	Acceptable	Acceptable
Were the effects of intervention reported comprehensively?	Yes	Yes	Yes	Yes	Yes	Yes	Yes
Was the precision of the estimate of the intervention or treatment effect reported?	Acceptable	Acceptable	Yes	Yes	Acceptable	Yes	Yes
Do the benefits of the experimental intervention outweigh the harms and costs?	Yes	Yes	Yes	Yes	Yes	Yes	Yes
Can the results be applied to your local population/in your context?	Yes	Yes	Yes	Yes	Yes	Yes	Yes
Would the experimental intervention provide greater value to the people in your care than any of the existing interventions?	Yes	Yes	Yes	Yes	Yes	Yes	Yes
Overall risk of bias	Low risk	Low risk	Low risk	Low risk	High risk	High risk	High risk
GRADE	Moderate	Moderate	High	Moderate	Low	Low	Low

[a]Can't tell cannot tell; criteria in this tool. Riedinger et al. [36], de Groot et al. [16], de Groot et al. [37], Omar et al. [38], Bauersfeld et al. [18], Dorff et al. [17], Zorn et al. [39].
