# Supplementary material for: Therapeutic Fasting in Reducing Chemotherapy Side Effects in Cancer Patients: A Systematic Review and Meta-Analysis
Source: Nutrients. 2023 Jun 8;15(12):2666. doi: 10.3390/nu15122666 (PMC10303481; doi:10.3390/nu15122666)
Supplement: Supplementary file 1 [file nutrients-15-02666-s001.zip › Table S2.pdf]

Table S2. Ongoing Clinical trials about Fasting regimens in cancer patients

Title	Study Type	Number participants	Study Start Date and Study End Date	Detailed Description (Aims)	ClinicalTrials.gov Identifier
DIRECT-2: Fasting Mimicking Diet Program to ImpRovE ChemoTherapy in HR+, HER2- Breast Cancer	Randomized Clinical Trial	240	May 1, 2023<br>May 1, 2032	Fasting Mimicking Diet during neoadjuvant chemotherapy aiming to improve the chemotherapy efficacy and decline the side effects in patients with stage II-III HR+, HER2- breast cancer	NCT05503108
Effect of Perioperative Dietary Restriction Strategy on Postoperative Recovery and Outcomes of Patients With Colorectal Cancer : a Multicenter Prospective, Randomized, Controlled Study	Randomized Clinical Trial	602	May 30, 2022<br>October 30, 2025	Evaluation of impact of fasting mimicking diet on postoperative recovery and outcomes of patients with colorectal cancer	NCT05384444
Intermittent Fasting Using a Fasting-Mimicking Diet to Improve Prostate Cancer Control and Metabolic Outcomes	Randomized Clinical Trial	138	June 30, 2023<br>June 30, 2029	Evaluation of the effects of a fasting mimicking diet (5 days per month eating L-Nutra products only for 6 months) vs. usual diet on response to cancer treatment of metastatic castrate sensitive prostate adenocarcinoma	NCT05832086
Exploiting Metformin Plus/Minus Cyclic Fasting Mimicking Diet (FMD) to Improve the Efficacy of First Line Chemo-immunotherapy in Advanced LKB1-inactive Lung Adenocarcinoma	Non Randomized Clinical Trial	64	October 30, 2018<br>September 10, 2023	Evaluation of the efficacy of combining standard-of-care platinum-based chemoimmunotherapy with metformin plus/minus Fasting Mimicking Diet in patients with LKB1-inactive, advanced lung adenocarcinoma	NCT03709147
Feasibility and Acceptability of Time Restricted Eating (TRE) Among Endometrial Cancer Patients: the TREND Study	Randomized Clinical Trial	15	October 7, 2021<br>September 2023	Evaluate the feasibility, fidelity and preliminary acceptability of TRE (among endometrial cancer patients, and to provide proof of principle that this dietary intervention can improve metabolic health in this population	NCT04783467
Time-Restricted Eating and Cancer: Clinical Outcomes, Mechanisms, and Moderators	Randomized Clinical Trial	300	January 1, 2022<br>December 2026	To test whether the timing of meals can improve treatment adverse events, influence tumor biology and alter a person's mood and behaviors	NCT04722341
The Safety and Efficacy of Time Restricted Eating Alone or Combined the Mediterranean Diet During Chemotherapy for Breast Cancer	Randomized Clinical Trial	40	August 1, 2022<br>March 1, 2026	To test the safety and feasibility of 8-h TRE compared to TRE combined with a mediterranean style diet among female breast cancer patients initiating chemotherapy for stage I-III breast cancer	NCT05259410
A Randomized, Phase II Clinical Trial of Time-Restricted Eating Versus Nutritional Counseling in Cancer Patients Receiving Radiation or Chemoradiation to Evaluate Its Impact on Toxicity and Efficacy	Randomized Clinical Trial	60	January 20, 2023<br>July 5, 2025	To determine if Time-Restricted Eating may improve the anti-cancer effects of radiation therapy and reduce the side-effects of this treatment in patients with prostate, cervical, and rectal cancers	NCT05722288
Time-Restricted Eating to Address Persistent Cancer-Related Fatigue: The Fatigue REDuction After Cancer (FREDA) Trial	Randomized Clinical Trial	30	January 26, 2023<br>April 2026	To assess feasibility of participants to adhere to Time-Restricted Eating and complete study activities. Evaluation if Time-Restricted Eating will lead to less fatigue at 12 weeks compared to the control	NCT05256888

Short-Term Fasting Prior to Standard Checkpoint Blockade Using PD-1/PD-L1 Inhibition: A Pilot Safety and Feasibility Study	Single Group	16	August 12, 2020<br>August 12, 2025	Undergoing short-term fasting prior to treatment with one of these PD-L1 or PD-1 inhibitors may potentially reduce the side effects of immunotherapy or even improve the effectiveness of immunotherapy in patients with skin malignancy	NCT04387084
Time Restricted Eating And Metformin (TEAM) in Invasive Breast Cancer (IBC) or Ductal Carcinoma in Situ (DCIS). A Randomized, Phase IIb, Window of Opportunity Presurgical Trial	Randomized Clinical Trial	120	April 4, 2023<br>November 17, 2025	Intermittent fasting may protect cancer patients from the toxic effects of chemotherapy agents without causing chronic weight loss. The combination of intermittent fasting and metformin may reduce breast cancer growth and may be used in women at risk for breast cancer or other cancers associated with being overweight	NCT05023967
