# Supplementary material for: Therapeutic Fasting in Reducing Chemotherapy Side Effects in Cancer Patients: A Systematic Review and Meta-Analysis
Source: Nutrients. 2023 Jun 8;15(12):2666. doi: 10.3390/nu15122666 (PMC10303481; doi:10.3390/nu15122666)
Supplement: Supplementary file 1 [file nutrients-15-02666-s001.zip › Table S3.pdf]

Table S3. PRISMA Checklist

Section and Topic	Item #	Checklist item	Reported on Page#
**TITLE**			
Title	1	**Therapeutic fasting regimens to reduce chemotherapy- side effects: A systematic review and meta-analysis**	1
**ABSTRACT**			
Abstract	2	**Importance: Studies on the potential impacts on cancer treatment from various forms of fasting, including the possibility that they reduce side effects, are still limited. Objective:** to determine the strength of the evidence on the effect of a variety of fasting-like regimens in preventing chemotherapy-related side effects. **Data Sources:** PubMed, Scopus and Embase were used to select the studies for this review ended on the 24th November 2022 according to Preferred Reporting Items for Systematic Reviews and Meta-Analyses guidelines. A combination of keywords as 'chemotherapy-related side effects' and 'fasting', 'fasting-mimicking diet' and other fasting regimens were used. The protocol was registered at the International Prospective Register of Systematic Reviews registry (CRD42022378131). **Study Selection:** All types of clinical trials and case series reporting chemotherapy toxicity on fasting regimens and any comparison. **Data Extraction and Synthesis:** Abstracts found to be relevant to this topic were shortlisted by two researchers. Details on the population characteristics, interventions, comparators and outcomes were extracted. Critical Appraisal Skills Programme checklists and the Grading of Recommendation, Assessment, Development and Evaluation approach were used for rating the certainty in evidence. Cochrane's Review Manager was used to perform a meta-analysis. **Main Outcome(s) and Measure(s):** Adverse Events grade over II and Neutropenia. **Results:** A total of 283 records were identified, of these, 274 were excluded, only 9 met inclusion criteria. Five of these trials were randomized. Overall, moderate/high quality of evidence shows that several fasting regimens did not have benefit when compared to conventional diet or any comparator in reducing the risk of adverse events. The overall pooled estimate for a variety of fasting regimes relative to a non-fasting one suggested no significant difference in the side effects (RR = 1.10; 95% confidence interval: 0.77-1.59; $I^2 = 10\%$, p=0.60) also considering only neutropenia (RR =1,33 ; 95% confidence interval :0.90-1.97 ; $I^2 = 0\%$, p=0.15). A sensitivity analysis confirmed these results. **Conclusions and Relevance:** Our systematic review and meta-analysis did not support, to date, the superiority of therapeutic fasting over non -fasting in preventing chemotherapy toxicity. The development of cancer treatment that are not associated with toxicities remains imperative.<br><br>https://www.crd.york.ac.uk/prospero/display_record.php?ID=CRD42022378131	2, 3
**INTRODUCTION**			

Section and Topic	Item #	Checklist item	Reported on Page#
Rationale	3	Describe the rationale for the review in the context of existing knowledge.<br><br>Based on all these evidence, fasting, although tumor-suppressive in healthy cells, may not exert beneficial effects in cancer cells. However, a variety of fasting regimens seems to be able in preventing the undesirable effects of chemotherapy but only few, small clinical studies have evaluated the potential of these dietary regimens as an adjunct to chemotherapy in reducing its toxicity.	4
Objectives	4	Provide an explicit statement of the objective(s) or question(s) the review addresses.<br>The objectives of this systematic review and meta-analysis are to determine the strength of the evidence on the effect of a variety of fasting-like regimens in cancer patients undergoing chemotherapy treatments in preventing chemotherapy-related side effects.	5
**METHODS**			
Eligibility criteria	5	Specify the inclusion and exclusion criteria for the review and how studies were grouped for the syntheses.<br>The inclusion criteria were as follows: 1) studies reporting chemotherapy-related toxicity on therapeutic fasting or fasting-like regimens in cancer patients; 2) all types of clinical trials and case series irrespective of participants' age and gender; 3) minimum sample size n=10; Although language was restricted to English, no cultural restrictions were placed on eligible studies during the search. Exclusion criteria were: pre-clinical studies, epidemiological studies, incidence and prevalence studies. Human studies including patients who were not receiving the standards of care for their malignancies were also excluded.	5, 6
Information sources	6	Specify all databases, registers, websites, organisations, reference lists and other sources searched or consulted to identify studies. Specify the date when each source was last searched or consulted.<br><br>The electronic databases PubMed (MEDLINE), Scopus, and Embase were used to select the studies included in this systematic review and meta-analysis. Search of articles ended on the 24th November 2022.	5
Search strategy	7	Present the full search strategies for all databases, registers and websites, including any filters and limits used.<br>A combination of the following keywords was used to search for literature:<br>('chemotherapy', 'chemotherapy toxicity', 'chemotherapy-related side effects') for population; ('fasting', 'fasting-mimicking diet', 'short-fasting' 'time-restricted eating', 'intermittent fasting') for intervention; The protocol was prospectively registered at the International Prospective Register of Systematic Reviews (PROSPERO) registry (CRD42022378131)<br><br>https://www.crd.york.ac.uk/prospero/display_record.php?ID=CRD42022378131	5, 3 supplement

Section and Topic	Item #	Checklist item	Reported on Page#
Selection process	8	Specify the methods used to decide whether a study met the inclusion criteria of the review, including how many reviewers screened each record and each report retrieved, whether they worked independently, and if applicable, details of automation tools used in the process.<br>Search results were imported into the reference manager software RefWorks (ProQuest) aiming at storing, organizing, and avoiding duplicates. Abstracts found to be relevant to the topic of interest were shortlisted by two researchers (OL and YF). Full-length papers of the shortlisted articles were assessed for the eligibility criteria. The articles that fulfilled the inclusion criteria were shortlisted for final systematic review. Eligible trials were read in full. The between authors disagreements were resolved through discussion among the authors until consensus was reached, otherwise a senior author (TM) arbitrated	6, 4 supplement
Data collection process	9	Specify the methods used to collect data from reports, including how many reviewers collected data from each report, whether they worked independently, any processes for obtaining or confirming data from study investigators, and if applicable, details of automation tools used in the process.<br>An electronic data sheet was developed and used for data extraction. Data were collected by two independent researchers. Allocation, randomization, blinding, comparability of the treatment and control groups, appropriateness and quality of the analysis performed as well as protocol registration and inclusion criteria pre-specification were verified rigorously by two researchers, to minimize bias and random error. An electronic data sheet was developed and used for data extraction. Data were summarized by all reviewers in a graphical summary.	6
Data items	10a	List and define all outcomes for which data were sought. Specify whether all results that were compatible with each outcome domain in each study were sought (e.g. for all measures, time points, analyses), and if not, the methods used to decide which results to collect.<br><br>The research questions were drawn using the PICO framework, which specifies the five key components of a well-defined therapeutic question: population, intervention, comparison, outcomes and study design. We extracted details on the population characteristics (eg, cancer patients, type of cancer, sample size), the intervention and comparator (eg, type of intervention i.e. fasting-mimicking diet- FMD, short-term fasting- STF and intermittent fasting- IF and any comparison) and the outcomes. The primary outcomes were Adverse Events (AEs) grade over II (considering AEs all together) and Neutropenia, according to the Common Terminology Criteria for Adverse Events (CTCAE) as reported in each study. We used a common summary outcome measure.	6
	10b	List and define all other variables for which data were sought (e.g. participant and intervention characteristics, funding sources). Describe any assumptions made about any missing or unclear information.<br>Publication date, n. patient, other reported outcomes. Bias due to missing results was avoided by consulting multiple bibliographic databases and trials registers, study authors or sponsors.	6, 7
Study risk of bias	11	Specify the methods used to assess risk of bias in the included studies, including details of the tool(s) used, how many reviewers assessed each study and whether they worked independently, and if applicable, details of automation tools used in the	7

Section and Topic	Item #	Checklist item	Reported on Page#
assessment		process.<br><br>CASP (Critical Appraisal Skills Programme) checklists were used as a tool for quality appraisal in health-related qualitative evidence syntheses; two researchers worked independently. The funnel plot was used as graphical device to detect study's precision and systematic heterogeneity	
Effect measures	12	Specify for each outcome the effect measure(s) (e.g. risk ratio, mean difference) used in the synthesis or presentation of results.<br><br>Dichotomous data were analyzed by calculating the risk ratio (RR) for each trial with 95% confidence intervals (CIs). We assessed heterogeneity of trial results by calculating a Chi-square test of heterogeneity and the I^2 measure of inconsistency. The main analysis was conducted using random-effects models	7
Synthesis methods	13a	Describe the processes used to decide which studies were eligible for each synthesis (e.g. tabulating the study intervention characteristics and comparing against the planned groups for each synthesis (item #5)).<br><br>studies reporting chemotherapy-related toxicity on therapeutic fasting in cancer patients;<br><br>clinical trials<br><br>case series irrespective of participants' age and gender.<br><br>works in English,<br><br>comparator: any comparatos as normal/usual diet or non dietary modification<br><br>The research questions were drawn using the PICO framework, which specifies the five key components of a well-defined therapeutic question: population, intervention, comparison, outcomes and study design. A research question was considered if all the elements of the PICO framework were provided and a conclusion about the direction of the effect was described anywhere in the SR. We extracted details on the population characteristics (eg, cancer patients, type of cancer), the intervention and comparator (eg, type of intervention i.e. STF, FMD, IF and any comparison) and the outcomes. The conclusions of the SR authors were classified into three categories: the "beneficial" category was used if there were conclusions with evidence of a positive effect; the "harmful" category was used when the reporting of the conclusions was clearly indicative of a harmful effect; the "no differential effect" category was used for conclusions that provided evidence for no difference between the intervention and the comparator.<br><br>We used our quality appraisal results to organise our thematic synthesis based on quality.	6, 7, 8
	13b	Describe any methods required to prepare the data for presentation or synthesis, such as handling of missing summary statistics, or data conversions.	6

Section and Topic	Item #	Checklist item	Reported on Page#
		Bias due to missing results was avoided by consulting multiple bibliographic databases and trials registers, study authors or sponsors.	
	13 c	Describe any methods used to tabulate or visually display results of individual studies and syntheses.<br><br>We reported in the tables and figures p value, CI and OR; heterogeneity;	7
	13 d	Describe any methods used to synthesize results and provide a rationale for the choice(s). If meta-analysis was performed, describe the model(s), method(s) to identify the presence and extent of statistical heterogeneity, and software package(s) used.<br><br>We performed a meta-analysis with Cochrane's Review Manager (RevMan) Software version 5.4. Heterogeneity was measured using the I^2 statistic, with values greater than 50% indicating substantial heterogeneity.	7
	13 e	Describe any methods used to explore possible causes of heterogeneity among study results (e.g. subgroup analysis, meta-regression).<br><br>The funnel plot was used as graphical device to detect study's precision and systematic heterogeneity.	7
	13f	Describe any sensitivity analyses conducted to assess robustness of the synthesized results.<br><br>The sensitivity analysis was carried out excluding non-randomised trial by RevMan version 5.4.	7
Reporting bias assessment	14	Describe any methods used to assess risk of bias due to missing results in a synthesis (arising from reporting biases).<br><br>Trials registers, study authors or sponsors were consulted.	7
Certainty assessment	15	Describe any methods used to assess certainty (or confidence) in the body of evidence for an outcome.<br><br>Pooled effect estimate from the meta-analysis was used for assessing the certainty in evidence. The GRADE (Grading of Recommendation, Assessment, Development and Evaluation) approach was used as framework for rating the certainty in evidence.	7
**RESULTS**			
Study	16	Describe the results of the search and selection process, from the number of records	

Section and Topic	Item #	Checklist item	Reported on Page#
selection	a	identified in the search to the number of studies included in the review, ideally using a flow diagram.	
	16b	Cite studies that might appear to meet the inclusion criteria, but which were excluded, and explain why they were excluded.	
Study characteristics	17	Cite each included study and present its characteristics.	Table 1

Source	Study size	Study design	Cancer type	Intervention treatment	Comparison	Outcomes
Riedinger et al, [30] 2020	24	RCT	Ovarian Uterine Cervical	STF 48 h - only water - for 24 h before and 24 h following each chemotherapy cycle.	No dietary modification (balanced, normo-caloric diet).	Chemotherapy related side effects and QOL.
de Groot et al, [16] 2015	13	RCT	Breast	STF 48 h - only water, coffee or tea without sugar - for 24 h before and 24 h following each chemotherapy cycle.	Normal diet according to the guidelines for healthy nutrition with a minimum of two pieces of fruit per day.	Feasibility and chemotherapy related side effects.
de Groot et al, [32] 2020	131	RCT	Breast	FMD 96 h - (plant-based low amino-acid substitution diet, consisting of soups, broths, liquids and tea) - for 3 days prior to and on the day of each chemotherapy cycle.	Normal diet.	Chemotherapy related side effects toxicity, radiological and pathological response to chemotherapy.
Omar et al, [31] 2022	48	RCT	Breast	IF 72 h - (fasting for 18 h from 12 am to 6 pm, with eating through 6 h	Normal diet.	Safety, practicality and chemotherapy related side effects toxicity.

Section and Topic	Item #	Checklist item							Reported on Page#
						from 6 pm to 12 am and with permission to drink water during fasting hours and eat small quantities of food vegetables, fruits, proteins and carbohydrates with a limitation of sugar and fats) - for the day before, during, and the day after chemotherapy.			
		Bauersfeld et al, [18] 2018	50	RCT	Breast Ovarian	STF 60 h - only water, herbal tea, and small standardized quantities of vegetable juice and of light vegetable broth – for 36 h before and 24 h following each chemotherapy cycle.	Normocaloric Mediterranean diet.	Safety and QOL	
		Dorff et al, [17] 2016	20	non-RCT	Breast Ovarian Uterine Urothelial NSCLC	STF 72 h – only water, non-caloric beverages, and small quantities of juice or food under 200 kcal/24 h – for	STF 24 h or 48 h – only water, non-caloric beverages, and small quantities of juice or food under		

Section and Topic	Item #	Checklist item	Reported on Page#

				48 h before and 24 h after chemotherapy.	200 kcal/24 h – for 24 h or 48 h before chemotherapy.	
Zorn et al, [30] 2020	51	non-RCT	Breast Endometrial Ovarian Cervical	mSTF 96 h – Ketogenic diet that provided between 400 and 600 kcal/day - for 72 h before and 24 h after chemotherapy	Normocaloric diet	Chemotherapy related side effects toxicity, QOL, fasting-related discomfort, compliance, nutritional status and laboratory parameters.
Lugtenberg et al, [33] 2021	131	RCT	Breast	FMD 96 h - (plant-based low amino-acid substitution diet, consisting of soups, broths, liquids and tea) - for 3 days prior to and on the day of each chemotherapy cycle.	Normal diet	QOL and illness perceptions.
Sadfie et al, [19] 2009	10	Case series	Breast Prostate Ovarian UterineNSCLC, Esophageal	Voluntarily fasted prior to 48-140 h and/or following 5-56 h chemotherapy	/	Chemotherapy related side effects toxicity

RCT, randomized control trial; STF, short-term fasting; mST, modified short-term fasting; FMD, fasting-mimicking diet; IF, intermittent fasting; QOL, quality of life; NSCLC, non-small-cell lung cancer; AEs, Adverse events.

Section and Topic	Item #	Checklist item	Reported on Page#
Risk of bias in studies	18	Present assessments of risk of bias for each included study. It was verified rigorously to minimise bias and random error, by two researchers, allocation, randomization and blinding, the comparability of the treatment and control	6, 7

Section and Topic	Item #	Checklist item	Reported on Page#
		groups, the appropriateness and quality of the analysis performed, and that a protocol was published and clear pre-specified inclusion criteria were present.<br><br>CASP (Critical Appraisal Skills Programme) checklists were used as a tool for quality appraisal in health-related qualitative evidence syntheses. Studies did not undergo critical appraisal because the inclusion criteria required a specific study design; thus, methodological differences were not present. If data from the same sample were presented in separate studies, the study with the largest sample size or the study examining more than one side effect was selected. If published data were insufficient to determine an effect size, studies were excluded.	
Results of individual studies	19	For all outcomes, present, for each study: (a) summary statistics for each group (where appropriate) and (b) an effect estimate and its precision (e.g. confidence/credible interval), ideally using structured tables or plots.<br><br>**Chemotherapy-side effects - GRADE > 2 (all studies)**<br>Study RR (95% CI)<br>de Groot 2015 1.71 [0.73, 4.03]<br>de Groot 2020 1.15 [0.92, 1.44]<br>Dorff 2016 0.46 [0.13, 1.62]<br>Omar 2022 0.33 [0.01, 7.80]<br>Riedinger 2020 0.20 [0.01, 3.70]<br>Zorn 2020 3.32 [0.14, 79.77]<br><br>**Chemotherapy-side effects - Neutropenia GRADE > 2 (all studies)**<br>Study RR (95% CI)<br>de Groot 2020 1.48 [0.98, 2.24]<br>Dorff 2016 0.74 [0.19, 2.89]<br>Omar 2022 0.33 [0.01, 7.80]<br>Riedinger 2020 0.33 [0.02, 7.32]<br><br>**Chemotherapy-side effects - GRADE > 2 (Sensitivity analysis)**<br>Study RR (95% CI)<br>de Groot 2015 1.71 [0.73, 4.03]<br>de Groot 2020 1.15 [0.92, 1.44]<br>Omar 2022 0.33 [0.01, 7.80]<br>Riedinger 2020 0.20 [0.01, 3.70]<br><br>**Chemotherapy-side effects - Neutropenia GRADE > 2 (Sensitivity analysis)**<br><br>Study RR (95% CI)<br>de Groot 2020 1.48 [0.98, 2.24]<br>Omar 2022 0.33 [0.01, 7.80]<br>Riedinger 2020 0.33 [0.02, 7.32]	Figure 2, Figure 3, Figure 4, eFigure 2
Results of	20	For each synthesis, briefly summarise the characteristics and risk of bias among	eTable

Section and Topic	Item #	Checklist item	Reported on Page#
syntheses	a	contributing studies.	1

Risk of bias assessment according to Critical Appraisal Skills Programme (CASP) for assessing randomized controlled trials

Criteria	Riedinger et al. 2020	de Groot et al. 2015	de Groot et al. 2020	Omar et al. 2022	Bauersfeld et al. 2018	Dorff et al. 2016	Zorn et al. 2020
Did the study address a clearly focused research question?	Yes	Yes	Yes	Yes	Yes	Yes	Yes
Was the assignment of participants to interventions randomised?	Yes	Yes	Yes	Yes	Yes	No	No
Were all participants who entered the study accounted for at its conclusion?	Yes	Yes	Yes	Yes	Yes	Yes	Yes
Were the participants 'blind' to intervention they were given?	No	No	No	No	No	No	No
Were the investigators 'blind' to the intervention they were giving to participants?	Can't tell[a]	Can't tell[a]	Yes	Can't tell[a]	Can't tell[a]	No	No
Were the people assessing/analysing outcome/s 'blinded'?	Can't tell[a]	Can't tell[a]	Yes	Can't tell[a]	Can't tell[a]	No	No
Were the study groups similar at the start of the randomised controlled trial?	Yes	Yes	Yes	Yes	No	No	No

Section and Topic	Item #	Checklist item								Reported on Page#
		Apart from the experimental intervention, did each study group receive the same level of care (that is, were they treated equally)?	Yes	Yes	Acceptable	Yes	Yes	Acceptable	Acceptable	
		Were the effects of intervention reported comprehensively?	Yes	Yes	Yes	Yes	Yes	Yes	Yes	
		Was the precision of the estimate of the intervention or treatment effect reported?	Acceptable	Acceptable	Yes	Yes	Acceptable	Yes	Yes	
		Do the benefits of the experimental intervention outweigh the harms and costs?	Yes	Yes	Yes	Yes	Yes	Yes	Yes	
		Can the results be applied to your local population/in your context?	Yes	Yes	Yes	Yes	Yes	Yes	Yes	
		Would the experimental intervention provide greater value to the people in your care than any of the existing interventions?	Yes	Yes	Yes	Yes	Yes	Yes	Yes	
		Overall risk of bias	Low risk	Low risk	Low risk	Low risk	High risk	High risk	High risk	
		[a]Can't tell cannot tell; criteria in this tool								
	20	Present results of all statistical syntheses conducted. If meta-analysis was done, present								10

Section and Topic	Item #	Checklist item	Reported on Page#
	b	for each the summary estimate and its precision (e.g. confidence/credible interval) and measures of statistical heterogeneity. If comparing groups, describe the direction of the effect.<br>The overall pooled estimate for a variety of fasting regimes relative to a non-fasting regime suggested no significant difference in the risk of chemotherapy-side effects from 6 single-center trial (RR = 1.10; 95% confidence interval [CI]: 0.77-1.59; I^2 = 10%, p=0.60) also considering only neutropenia (4 trials, RR =1,33 ; 95% confidence interval [CI]:0.90-1.97 ; I^2 = 0%, p=0.15) (16,31-33).	
	20 c	Present results of all investigations of possible causes of heterogeneity among study results.<br><br>The funnel plot was used as graphical device to detect study's precision and systematic heterogeneity	6, eFigure 1
	20 d	Present results of all sensitivity analyses conducted to assess the robustness of the synthesized results.<br>The sensitivity analysis, excluding non-randomised trial (17,30), resulted in no differences in the odds ratios and significance of results with regards to the prespecified endpoint (in 4 RCT, total toxicity: RR = 1.16; 95% confidence interval [CI]: 0.93-1.44; I^2 = 0%, p=0.18; in 3 RCT, neutropenia RR=1.40, 95% confidence interval [CI]: 0.93-2.11; I^2 = 0%, p=0.10).	10
Reporting biases	21	Present assessments of risk of bias due to missing results (arising from reporting biases) for each synthesis assessed.<br><br>The methodological quality of the studies is shown in eTable 1 (in the Supplement). A total of 4 studies was rated as at low risk of bias [16, 30-32] . Allocation concealment and selective reporting were appropriate in these studies. All studies had no risk of bias for incomplete reporting. The overall quality was assessed and rated as high risk of bias for three studies. [17,18, 34]<br><br>Data from a same sample was presented in two separate studies,[32, 33] therefore only the study examining more side effect was analyzed.[32]<br><br>Since in the study of Bauersfeld et al.[18] there were no toxicity events grade III/IV adverse events documented, and in two studies [16, 31,] none of the participants developed neutropenia, we excluded these works from the estimation of the risk of chemotherapy-side effects in the overall pooled estimate and in the neutropenia pooled estimate, respectively	7, 9, eTable 1
Certainty of evidence	22	Present assessments of certainty (or confidence) in the body of evidence for each outcome assessed.<br>None of the interventions tested resulted beneficial (according to main outcomes) and all were classified as having “no differential effect” (Table 1).	9, Table 1, eTable 1

Section and Topic	Item #	Checklist item	Reported on Page#
		Overall, moderate/high quality of evidence shows that several fasting regimens did not have benefit when compared to conventional diet or any comparator in reducing the risk of AEs (eTable 1).	
**DISCUSSION**			
Discussion	23a	Provide a general interpretation of the results in the context of other evidence.<br><br>Our systematic review did not show any clear effect of several fasting-like regimens in reducing chemotherapy-related side effects as nausea, vomiting, weakness and hematologic toxicity (analyzed all together, Table 1), which severely impact the quality of life of cancer patients undergoing chemotherapy.<br><br>The current meta-analysis did not demonstrate superiority of therapeutic fasting (STF, /FMD / IF; patients n=210) over non -fasting in preventing chemotherapy-related side effects when evaluating both randomised and non-randomised studies together (Figure 2 and 3). After a quality appraisal, we included only 4 RCT in the sensitivity analysis. We did not find a significant difference in AEs rate (grade III/IV) and neutropenia (only 3 RCT) between participants on fasting-like regimens and non-fasting people (p= 0.18 and p=0.10 respectively, Figure 4 and 5), though individual studies reported lower rates of AEs for fasting-like people<br><br>The results from this review are comparable to a work by Caccialanza et al. (9) which synthesized most of the available studies regarding fasting or calorie restriction in cancer patients. Fasting or calorie restriction did not constantly show anticancer effects in preclinical studies (10-15). Furthermore, the evidence provided by preclinical and human studies on the beneficial effect of a variety of fasting regimens on chemotherapy-related side effects is still very limited.	2, 10, 11
	23b	Discuss any limitations of the evidence included in the review.<br><br>The results of this meta-analysis should be interpreted cautiously, as some studies were heterogeneous and sample sizes were low and variable. The contribution of age, co-morbidities and polypharmacy to the outcome remain difficult to assess. Taken together, these attributes make the conclusions arising from our meta-analysis predominately hypothesis-generating rather than definitive and/or conclusive. It cannot be excluded that in the future, if larger studies will be carried out, completely different results from those reported here will not be obtained.	13
	23c	Discuss any limitations of the review processes used.<br>Studies that were not published in English were excluded. However, the inclusion of qualitative literature provides a richness to the data synthesis.	13
	23d	Discuss implications of the results for practice, policy, and future research.<br><br>One must be cautious in the application of the clinical trials testing the effect of fasting or other fasting regimes in preventing chemotherapy-related side effects in cancer patients. The careful analysis of these clinical trials did not show any clear association	13

Section and Topic	Item #	Checklist item	Reported on Page#
		between these fasting regimens and the reduction of the most common side effects as nausea, vomiting, weakness, gastrointestinal side effects and hematologic toxicity. We would argue that the role of fasting as a mechanism useful in reducing the chemotherapy-side effects remains unresolved and all this arguing seems *much ado about nothing* (9). For the moment, food restriction before and/or after chemotherapy does not have sufficient scientific basis to be prescribed by an oncologist or nutritionist. This review may allow discussing on an area that received little attention in the literature until now, which may represent potential themes for future research. Future studies examining pathogenesis and effects of treatment are badly needed.<br><br>Although there is limited evidence on the association between chemotherapy-related side effects and general dietary recommendations, it is likely the individualized approach of counselling, that considers patients preferences, symptoms and medical status, is a key element in improving the side effects of chemotherapy	
**OTHER INFORMATION**			
Registration and protocol	24a	Provide registration information for the review, including register name and registration number, or state that the review was not registered.<br>(PROSPERO) registry (CRD42022378131);	3, 5
	24b	Indicate where the review protocol can be accessed, or state that a protocol was not prepared.<br>https://www.crd.york.ac.uk/prospero/display_record.php?ID=CRD42022378131	5
	24c	Describe and explain any amendments to information provided at registration or in the protocol.<br>no amendment	
Support	25	Describe sources of financial or non-financial support for the review, and the role of the funders or sponsors in the review.<br>no sponsor<br><br>This research received no specific grant from any funding agency in the public, commercial, or not-for-profit sectors	14
Competing interests	26	Declare any competing interests of review authors.<br>The authors declare that they have no known competing financial interests or personal relationships that could have appeared to influence the work reported in this paper.	14
Availability of data, code and other materials	27	Report which of the following are publicly available and where they can be found: template data collection forms; data extracted from included studies; data used for all analyses; analytic code; any other materials used in the review.<br><br>on request; Supplemental materials	Supplement
